# Elucidating ascorbate and aldarate metabolism pathway characteristics via integration of untargeted metabolomics and transcriptomics of the kidney of high-fat diet-fed obese mice

**DOI:** 10.1371/journal.pone.0300705

**Published:** 2024-04-11

**Authors:** Hong Liang, Kang Song

**Affiliations:** 1 Department of Basic Medical Sciences, Medical College, Qinghai University, Xining, Qinghai, China; 2 Endocrinology Department, Qinghai Provincial People’s Hospital, Xining, Qinghai, China; 3 Qinghai University Affiliated People’s Hospital, Xining, Qinghai, China; Zagazig University, EGYPT

## Abstract

Obesity is a major independent risk factor for chronic kidney disease and can activate renal oxidative stress injury. Ascorbate and aldarate metabolism is an important carbohydrate metabolic pathway that protects cells from oxidative damage. However the effect of oxidative stress on this pathway is still unclear. Therefore, the primary objective of this study was to investigate the ascorbate and aldarate metabolism pathway in the kidneys of high-fat diet-fed obese mice and determine the effects of oxidative stress. Male C57BL/6J mice were fed on a high-fat diet for 12 weeks to induce obesity. Subsequently, non-targeted metabolomics profiling was used to identify metabolites in the kidney tissues of the obese mice, followed by RNA sequencing using transcriptomic methods. The integrated analysis of metabolomics and transcriptomics revealed the alterations in the ascorbate and aldarate metabolic pathway in the kidneys of these high-fat diet-fed obese mice. The high-fat diet-induced obesity resulted in notable changes, including thinning of the glomerular basement membrane, alterations in podocyte morphology, and an increase in oxidative stress. Metabolomics analysis revealed 649 metabolites in the positive-ion mode, and 470 metabolites in the negative-ion mode. Additionally, 659 differentially expressed genes (DEGs) were identified in the obese mice, of which 34 were upregulated and 625 downregulated. Integrated metabolomics and transcriptomics analyses revealed two DEGs and 13 differential metabolites in the ascorbate and aldarate metabolic pathway. The expression levels of *ugt1a9* and *ugt2b1* were downregulated, and the ascorbate level in kidney tissue of obese mice was reduced. Thus, renal oxidative stress injury induced by high-fat diet affects metabolic regulation of ascorbate and aldarate metabolism in obese mice. Ascorbate emerged as a potential marker for predicting kidney damage due to high-fat diet-induced obesity.

## Introduction

Obesity is a complex disease with significant public health implications worldwide [1]. Obesity is caused by many factors, including diet, lack of physical activity, environmental factors, and genetic [2].Numerous studies have established the detrimental impact of high-fat diets on human health, indicating a positive correlation with obesity [3, 4]. Obesity is a risk factor for many metabolic diseases, such as type 2 diabetes mellitus and non-alcoholic fatty liver disease [5–7]. It is also a major independent risk factor for chronic kidney disease [8].

Kidneys are essential for normal body function [9] that mainly regulate fluid, electrolytes, and the acid-base balance in the body to create a stable environment for tissue and cellular metabolism. Long-term high-fat diet feeding affects kidney energy balance in mice, leading to lipid accumulation and impaired mitochondrial function [10], which, in turn, leads to obesity and metabolic syndrome, further causing changes in kidney lipid metabolism and renal injury [11, 12]. In many countries, typical diets have a high fat content, thereby increasing the risk of non-communicable diseases, including renal disorders. Research has shown that obese patients are at higher risk of developing chronic kidney disease compared to metabolically healthy normal-weight individuals, independent of metabolic parameters [13].

Obesity is associated with increased oxidative stress and excessive amount of reactive oxygen species (ROS) in cells and tissues [14]. Obesity can induce oxidative stress by disrupting the adipose tissue microenvironment, thereby mediating mild chronic inflammation and mitochondrial dysfunction [15] and disrupting homeostasis. Aascorbate and aldarate metabolism is an important carbohydrate metabolic pathway that protects cells from oxidative damage [16]. Studies in humans and rats have revealed low ascorbate levels in diabetic nephropathy [17, 18]. However, changes in ascorbate and aldarate metabolism in kidney tissues mediated by high-fat diet induced obesity have not been reported.

Metabolomics provides a comprehensive molecular profile of a specific phenotype by integrating information from both endogenous and exogenous sources [19], thereby providing an intuitive and accurate representation of the physiological state. Contrarily, the transcriptome serves as a link between genetic information and biological function, making it a crucial tool for evaluating gene expression. Farmanullah *et al*. [20] used advanced bioinformatics methods such as hierarchical clustering, enrichment analysis, active site prediction, and functional domain identification through transcriptomics to analyze important genes that could be used as biomarkers and therapeutic targets for mastitis. Talpur *et al*. [21] demonstrated, using a weighted Gene Co-Expression Network Analysis (WGCNA) algorithm that relevant modules and key gene drivers associated with external traits can be determined, further identifying differentially expressed genes (DEGs). Integrating transcriptomics and metabolomics data allows for normalization and statistical analyses across different biomolecular levels, facilitating the exploration of molecular relationships between these levels. By employing biological function analysis, metabolic pathway enrichment, and other methods, a systematic and comprehensive analysis of biomolecular functions and regulatory mechanisms can be performed, which can further lead to identification of key metabolic pathways. Data integration often involves aligning data with corresponding Kyoto Encyclopedia of Genes and Genomes (KEGG) pathways. Guo *et al*. [22] successfully utilized combined transcriptomic and metabolomic analyses to understand the molecular mechanisms underlying the effects of drugs on acute kidney injury. Overall, integrating multiple omics data can help identify previously unrecognized interrelationships between different biological or molecular pathways. Accordingly, studies have used multi-omics analyses to uncover the pathophysiological mechanisms and potential biomarkers of diabetic nephropathy [23, 24] to elucidate the relationship between obesity and kidney damage.

In the present study,we utilized an ultra-high-performance liquid chromatography system to analyze changes in kidney metabolites in obese mice and performed RNA sequencing to identify genes that are differentially expressed in kidney tissues of mice fed with a high-fat diet compared to those of mice fed with a normal diet. By integrating differentially expressed metabolites and genes, we aimed to investigate the metabolic changes in the ascorbate and aldarate metabolism pathway in obese mice under oxidative stress injury and screen biological markers of kidney injury in obese mice.

## Materials and methods animal models

**Fig 1:**
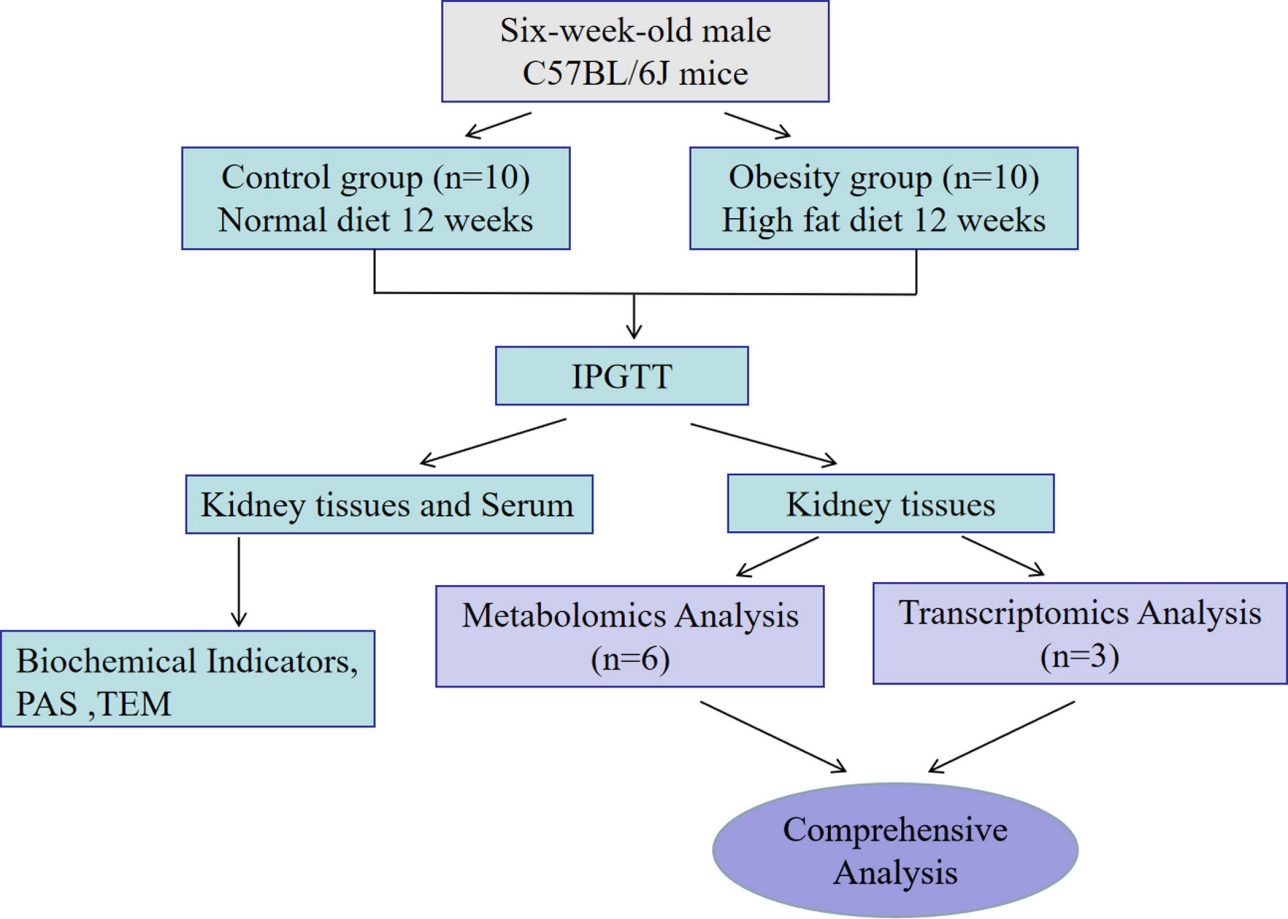


Twenty male C57BL/6J 6 weeks mice were procured from Chengdu Dossy Experimental Animal Co. Ltd. (Chengdu, China). These mice were randomly divided into two groups (n = 10 per group): a control group and an obesity group. The control group received a regular diet, whereas the obese group was fed a high-fat diet (60 kcal% fat, Research diet D12492) [25, 26] for 12 weeks. The mice were housed in a specific pathogen-free animal laboratory under controlled temperature (22 ± 2°C) and humidity (55 ± 5%). Throughout the experiment, both groups had unrestricted access to food and water, and their weights were recorded weekly. This study was approved by the Ethics Committee of the Qinghai Provincial People’s Hospital (ethical approval number: 2023–026), and all animal experiments adhered to China’s Experimental Animal Management Regulations. After 12 weeks, all mice were euthanized using sodium pentobarbital (Sigma-Aldrich; 50 mg/kg) [27–29] and sacrificed for further study. The study design is shown in Fig 1.

### Intraperitoneal glucose tolerance test

Prior to the glucose tolerance test, the mice underwent a 12-h fasting period. Subsequently, they received an intraperitoneal injection of 1.5 g/kg glucose. Thereafter, blood samples were collected from the tail of the mice at 0, 15, 30, 60, and 120 min, and glucose levels were measured using an automatic glucose monitor (Xinjiang Hengsheng Biological Co. Ltd., Urumchi, China).

### Serum and tissue samples

Blood was collected from the inner canthal vein [30] and kidney tissues were collected from the mice after overnight fasting and euthanasia. Paraffin-embedded sections of kidney tissue fixed in 4% paraformaldehyde were used for histological studies, whereas kidney specimens fixed in glutaraldehyde were subjected to electron microscopy. Omics and related indices were determined using kidney tissues fixed in liquid nitrogen and preserved at -80°C. Additionally, white adipose tissue from the epididymis and perirenal was collected and weighed.

### Biochemical indicators detection

(1) Commercial kits A042-1 from Nanjing Jiancheng Bioengineering Institute (Nanjing, China) were used to measure serum non-esterified fatty acids (NEFA). (2) Commercial kits A111-2-1, A110-1-1, A001-3, A003-1, and A007-2-1 from Nanjing Jiancheng Bioengineering Institute (Nanjing, China) were used to detect total cholesterol (TCH) in the kidney tissue, triglyceride (TG), superoxide dismutase (SOD), malondialdehyde (MDA), and catalase (CAT) activities, respectively.

### Periodic Acid Schiff (PAS) staining

Fixed kidney tissue from the mice were subjected to dehydration, pruning, embedding, slicing, and staining with PAS. The stained tissue sections were examined and scanned using a Pannoramic 250 digital slide scanner (3DHISTECH, Hungary). The Image-Pro Plus 6.0 image analysis system (Media Cybernetics Inc, Maryland, USA) was used to determine the positive expression area in the collected images; the positive expression area percentage was calculated by dividing the positive expression area by the visual field area (pixels).

### Transmission electron microscopy (TEM)

Kidney renal cortex tissues fixed in 3% glutaraldehyde and 1% osmium tetroxide were subjected to dehydration using acetone. Subsequently, tissues were embedded in Epon 812, and ultrathin sections (approximately 60 nm) were cut using a ultramicrotome. The sections were placed in copper grids, stained with lead citrate, and examined using an electron microscope (JEOL JEM-1400,Japan electronics co. LTD, Japan).

### Untargeted metabolomics analysis

After the mice had been euthanized, kidney tissues were fixed in liquid nitrogen and preserved at -80°C. A cold extraction solvent methanol/acetonitrile/H_2_O (2:2:1, *v*/*v*/*v*) was added (to make up the volume to 1 mL) to 80 mg samples (the same location) to extract metabolites from kidney tissue samples. The samples were separated on a ACQUIY UPLC BEH Amide column (2.1 mm × 100 mm, 1.7μm particle size(waters, Ireland) [31] with the following parameters: Column temperature 25°C; flow rate 0.5mL/min; sample size 2 L; mobile phase composition A: water + 25 mM ammonium acetate + 25 mM ammonia water, B: acetonitrile; The gradient exit procedure was as follows: 0–0.5 min. 95%B; at 0.5-7min, B changed linearly from 95 to 65%; 7–8 min, B changed linearly from 65 to 40%; 8–9 min, B maintained at 40%; 9–9.1 min, B changed linearly from 40 to 95%; 9.1–12 min, B remained at 95%. The samples were placed in four automatic samplers during the entire analysis process. The samples were analyzed continuously in random order. Quality control (QC) samples were inserted into the sample queue to monitor the stability of the species evaluation system and the reliability of the experimental data. Mass spectrometry was performed using a TripleTOF 6600 spectrometer (SCIEX, USA) in both negative and positive ionization modes [32]. The original data was converted into mzXML format using ProteoWizard, and then XCMS software was used for peak alignment, retention time correction, and peak area extraction. First, the metabolite structure identification and data preprocessing were performed for the data extracted by XCMS, followed by experimental data quality evaluation and data analysis.

In this study, In-house Database (Shanghai Applied Protein Technology) was used to search [32, 33]. By matching the information of metabolite retention time, molecular mass (molecular mass error within 25 ppm), secondary fragmentation spectrum, collision energy, and other information in the local database, the structure of metabolites in biological samples was identified, and the identification results were checked stringently and confirmed manually. The identification level was above Level 2. Significance was determined using an unpaired Student’s *t*-test. A variable importance for the projection (VIP) value > 1 and *P* < 0.05 were considered statistically significant.

### Transcriptome analyses

#### RNA extraction

In this study, TRIzol reagent (Magen, Guangzhou, China) was used to extract RNA from kidney tissue [34]. Thereafter, the A260/A280 absorbance ratio was examined using a NanoDrop spectrophotometer (Thermo Scientific, USA). RNA quality (RNA integrity number) was measured using an Agilent Bioanalyzer 4150 (Agilent Technologies, CA, USA).

### Library construction and sequencing

High-quality RNA samples were used to construct an RNA library. Magnetic beads with oligo (dT) bound to their surface were used for mRNA purification. Fragmentation buffer was added to mRNA to initiate random fragmentation. Six bases random hexamers were then used to synthesize cDNA using mRNA as a template. Buffer, dNTPs, and DNA polymerase I were then added to synthesize the second strand cDNA. The synthesized double-stranded cDNA fragment was connected to the splice sequence for Polymerase Chain Reaction (PCR) amplification. PCR products were purified and library quality was assessed using Agilent Bioanalyzer 4150 (Agilent Technologies Inc. California, USA). This was performed using the NovaSeq 6000 (Illumina, California, USA) sequencing platform PE150 read length.

### RNA-seq data analyses

The data generated from the Illumina platform was used for bioinformatics analyses. The mapped reads for subsequent analysis were obtained by comparing the clean reads to the reference genome using the HISAT2 software (http://daehwankimlab.github.io/hisat2/) [35]. Expected number of Fragments Per Kilobase of transcript sequence per Millions base pairs sequenced (FPKM)values for each gene in each sample were calculated using the featureCounts software. Use DESeq2 (http://bioconductor.org/packages/release/bioc/html/DESeq2.html) [36] for group differences between gene expression analysis.The screening threshold of differentially expressed genes was | log_2_FC |>1 and *P*≤0.05. The ClusterProfiler R software package was used for Gene Ontology (GO)functional enrichment and KEGG pathway enrichment analysis [37]. Results with *P* ≤ 0.05 were considered significant for enrichment of GO or KEGG function.

### Network analysis of metabolomics and transcriptomic data

DEGs and metabolites were collated and summarized for KEGG enrichment and network analysis. Data analysis was performed using the R package (version 3.6.3) Cytoscape (version 3.8.2) to investigate interactions between identified genes and metabolites.

### Statistical analyses

Data were analyzed using GraphPad Prism 8.0 (GraphPad Software, San Diego, CA, USA). The *t*-test was used for comparing independent samples of continuous data, in which data are presented as the mean ± standard deviation of the mean (SD). Results with *P* < 0.05 were considered statistically significant.

## Results

### Effects of a high-fat diet on body weight and glucolipid metabolism

**Fig 2:**
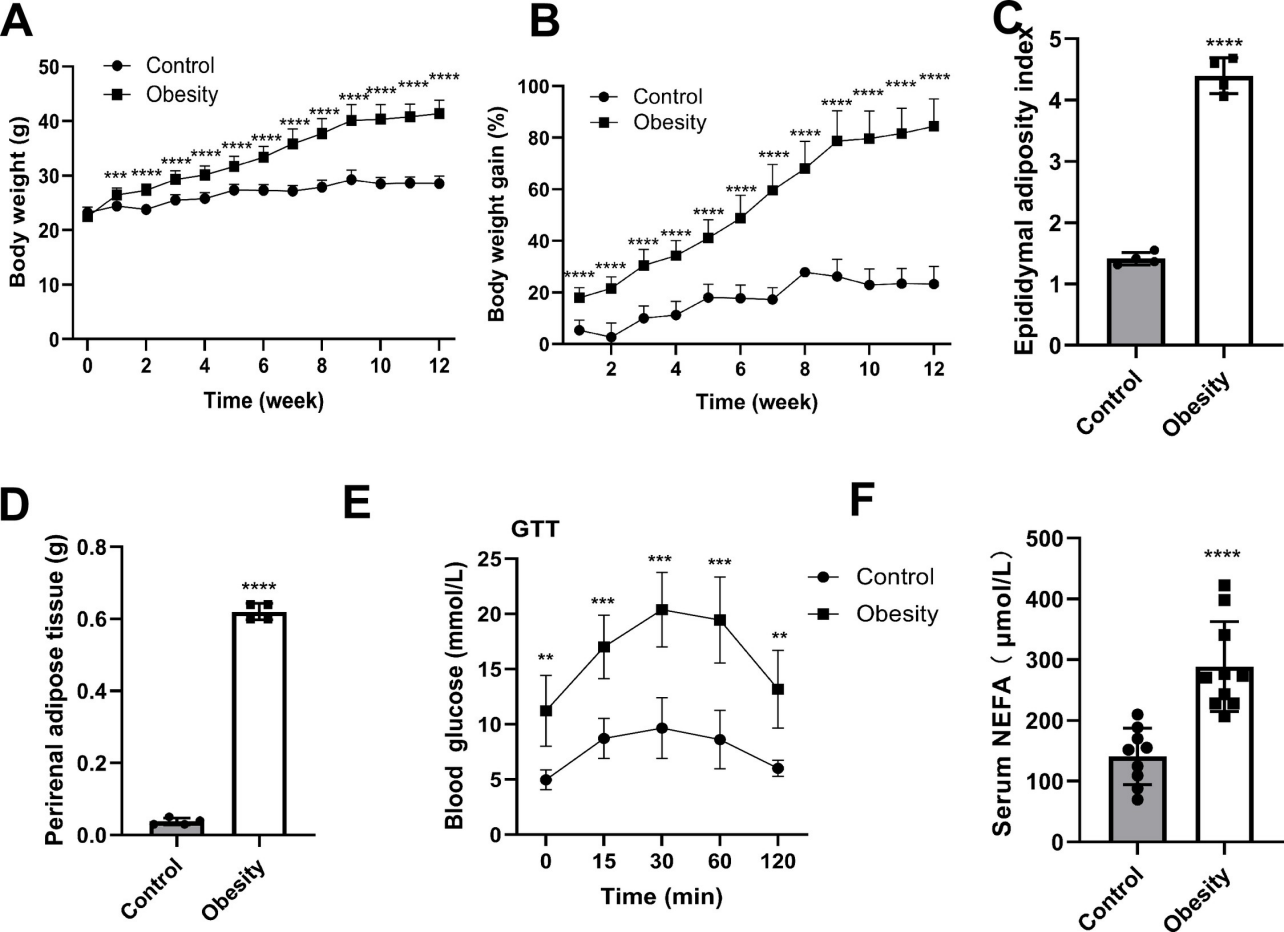
Effects of a high-fat diet on body weight and metabolism of glucolipids in obese mice. (A) Changes in body weight over 12 weeks; (B) Changes in relative body weight gain in mice; (C) Epididymal adiposity index; (D) Perirenal adipose tissue; (E) Glucose tolerance test (GTT); (F) Serum NEFA. A and B, n = 10 each group; C and D, n = 4 each group; E,n = 5 each group; F,n = 9–10 each group.**P < 0.01, ***P < 0.001,****P < 0.0001 *vs* Control group.

During the 12-week high-fat diet-fed to mice, we observed an effect on the body weight. Compared with the control group, the obese group showed a significant increase in body weight, starting from high-fat diet intake (Fig 2A, *P* < 0.05). At 9 weeks, the average weight of the obese mice (40 g) was 137% higher than that of the control mice. Overall, the obese mice gained 78.7% more weight compared to the baseline, while the control group gained only 26.2% more weight (Fig 2A and 2B, *P* < 0.05). Following the 12-week period, the obese mice averaged 41.4 g, whereas the control mice averaged 28.6 g (Fig 2A, *P* < 0.05). To further investigate the distribution of adipose tissue in the two groups, we weighed the perirenal adipose tissue and calculated the epididymal adiposity index. Obese mice had a significantly higher epididymal adiposity index and perirenal adipose tissue compared to the control mice (Fig 2C and 2D, *P* < 0.05). High-fat diets can dysregulate glucose and lipid metabolism. Expectedly, the glucose levels in the obesity group were consistently higher than those in the control group as determined by the glucose tolerance test. Furthermore, obese mice exhibited reduced glucose clearance compared to control mice (Fig 2E). Additionally, the serum levels of NEFA were considerably higher in the obese group than in the control group (Fig 2F).

### Renal changes in obese mice

**Fig 3:**
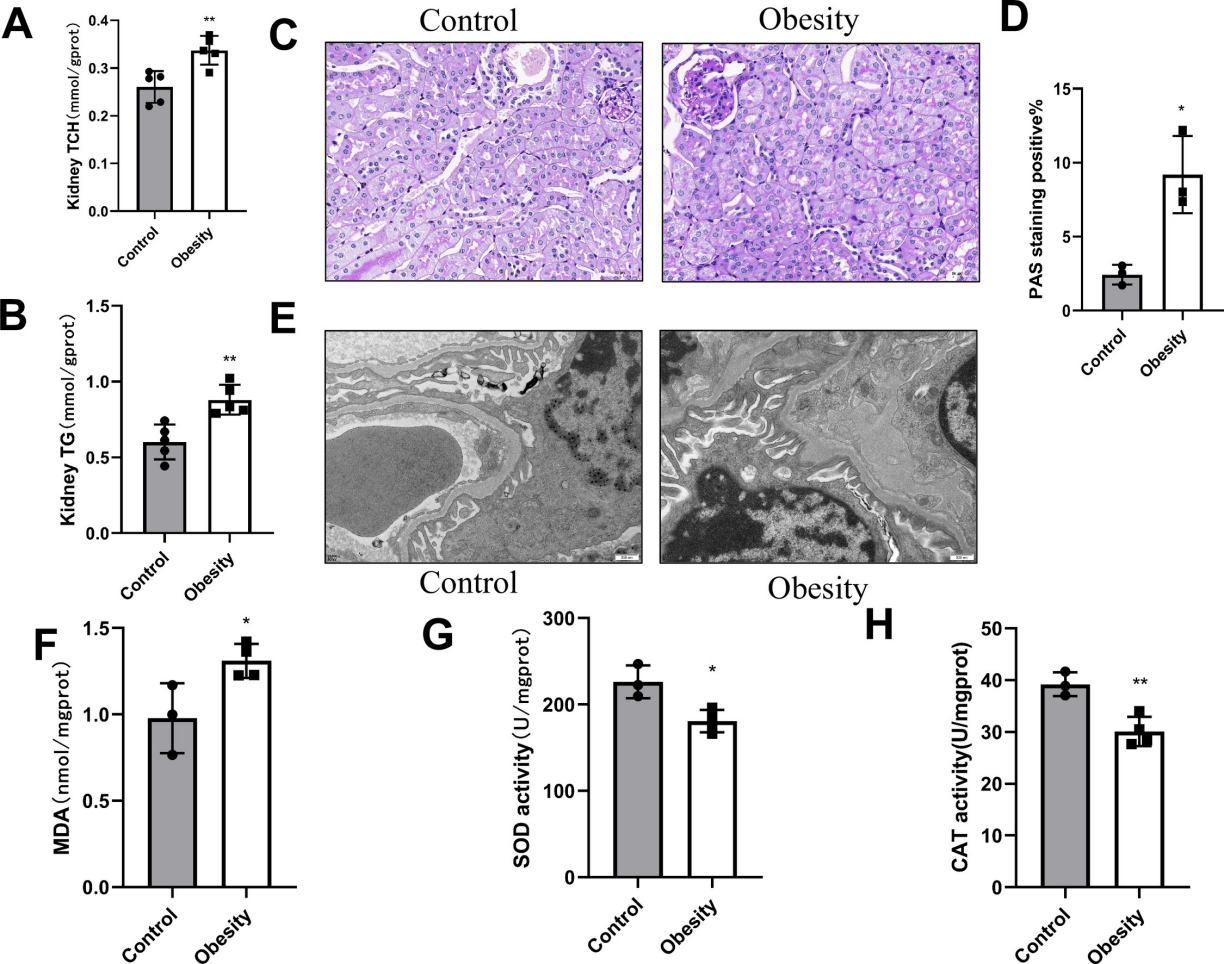
Renal changes in obese mice induced by high-fat diet obesity mice. (A) The content of kidney total cholesterol (TCH) and (B) kidney triglyceride (TG), **P < 0.01 versus Control group, n = 5 each group. (C) Sections of kidney tissue stained with PAS, magnification, 400×, and (D) The percentage of renal tissue stained with PAS, *P < 0.05 versus Control group, n = 3 each group. (E) Transmission electron microscopy of renal ultramicrostructure, magnification, 25000×, n = 3 each group. (F) Malondialdehyde (MDA) content, superoxide dismutase(SOD)activity, catalase (CAT) activity, *P < 0.05, **P < 0.01, n = 3–4 each group.

Quantification of lipids extracted from the kidneys demonstrated a substantial increase in the cholesterol and triglyceride content in the obese mice compared to the control group (Fig 3A and 3B). Ectopic accumulation of lipids in the kidneys cause lipotoxicity and subsequent renal injury. Therefore, PAS staining was performed to assess glycogen accumulation in kidney tissues. A considerable increase in PAS-positive areas was observed in the obesity group compared to the control group, indicating increased glycogen accumulation in the kidneys of obese mice (Fig 3C and 3D). To investigate the effects of high-fat diet on kidney ultrastructure, TEM was performed. Obese mice showed altered renal ultrastructure, with thinning of the glomerular basement membrane (GBM) and blurred edges, irregular thickness, and foot process fusion. Moreover, abnormal morphology and structure of podocytes, swollen and fractured mitochondria, and flocculants in the matrix were observed (Fig 3E). In contrast, the control group exhibited normal GBM thickness and intact and ordered foot processes (Fig 3E). Additionally, measurements of oxidative stress markers in the kidney revealed a considerable reduction in SOD and CAT activity and a considerable increase in MDA levels in obese mice (Fig 3F–3H).

### Renal metabolomic changes in obese mice

**Fig 4:**
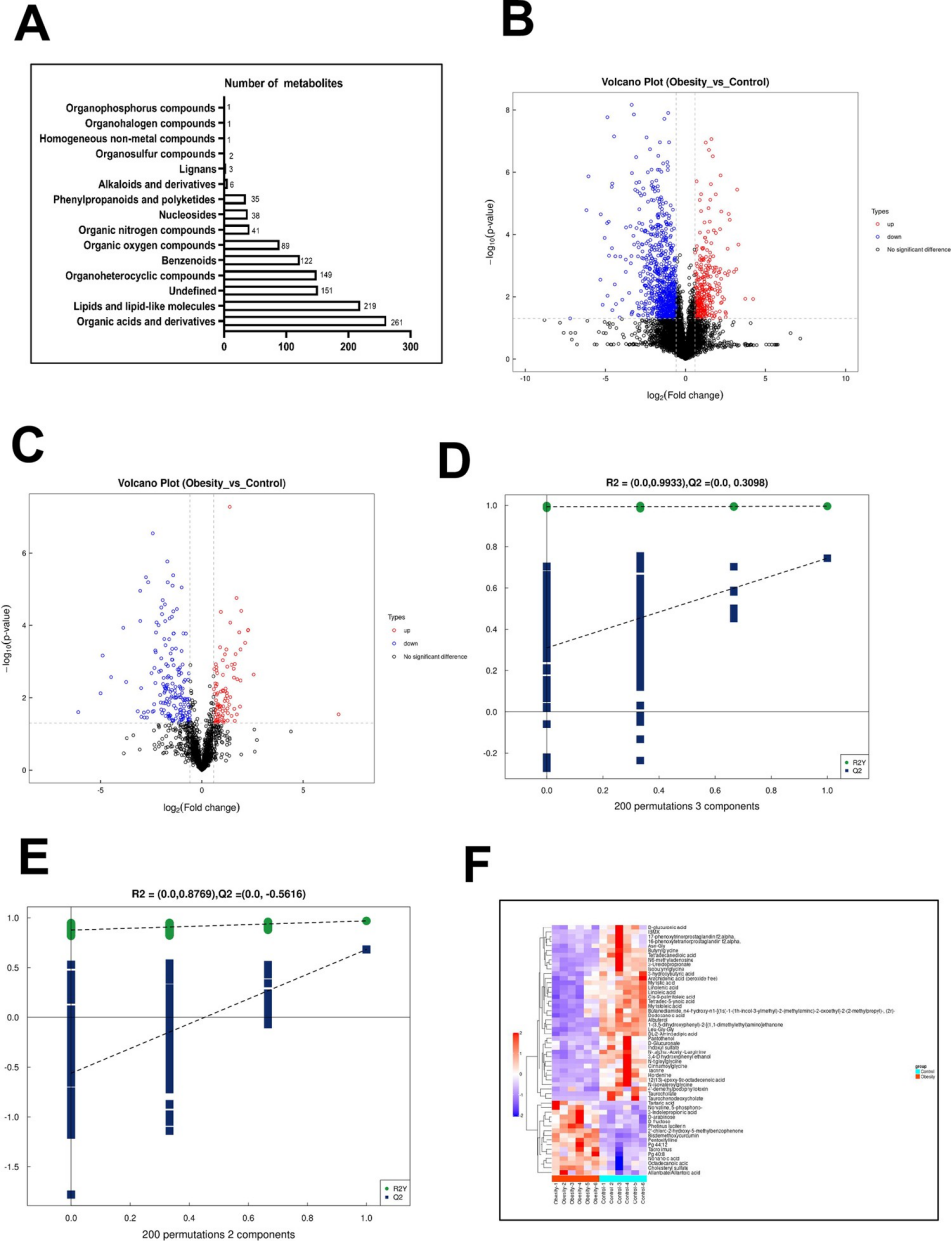
Changes in metabolomics of renal tissue in high-fat diet obesity mice. (A) Both groups identified different types and quantities of metabolic compounds. Positive and negative ion volcano plots (B, C), the blue and red round dots indicate metabolites that are downregulated and upregulated. (D) Positive and (E) negative ion partial least squares discrimination analysis (PLS-DA) permutation test. (F) The hierarchy of clustering of differential metabolites in negative ion modes. A-F, n = 6 each group.

Untargeted metabolomic analysis in both positive and negative ion spectrophotometer modes identified 649 and 470 metabolites, respectively. The majority of these metabolites were classified as organic acids and organic acid derivatives, followed by lipids and lipid-like molecules (Fig 4A). All differentially expressed metabolites were identified in the positive and negative ion modes, with FC > 1.5 or FC < 0.67 (*P* < 0.05) as the standard, and are shown as a volcanic map (Fig 4B and 4C). In total, 314 metabolites were upregulated and 849 were downregulated in the positive ion mode, while 91 were upregulated and 171 were downregulated in the negative ion mode (Fig 4B and 4C). Permutation testing confirmed the reliability of the model. In principal component analysis (PCA), principal component (PC)1explained 21.2%, PC2 17.6%, and PC3 12.9% of the variability in the data analyzed in positive ion model and PC1 explained 24.8%, PC2 20.3% and PC3 15% of the variability in data analyzed in negative ion mode, as shown in S1-1 and S1-2 Figs in S1 File. The degree of aggregation within the group and the separation between the groups were obvious, indicating the reliability of the model. For the positive and negative ion modes, Fig 4D and 4E indicate downward trends in R2 and Q2, respectively, with decreasing replacement retention, indicating the reliability of the model. A total of 13 metabolites participated in the ascorbate and aldarate metabolism pathway, as shown in S2-1 Table in S2 File. To comprehensively evaluate the relationship between samples and identify differences in metabolite expression patterns, we performed cluster analysis. Metabolites with significant differences (VIP > 1, *P* < 0.05) were displayed on a heat map and showed clustering based on similar expression patterns, thereby indicating potential functional activity or participation in the same metabolic pathways. As shown in Fig 4F, the expression of D-Glucuronate and D-arabinose,the metabolites involved in the ascorbate and aldarate metabolism pathway, changed in the kidneys of obese mice.

### Renal transcriptomic changes in obese mice

**Fig 5:**
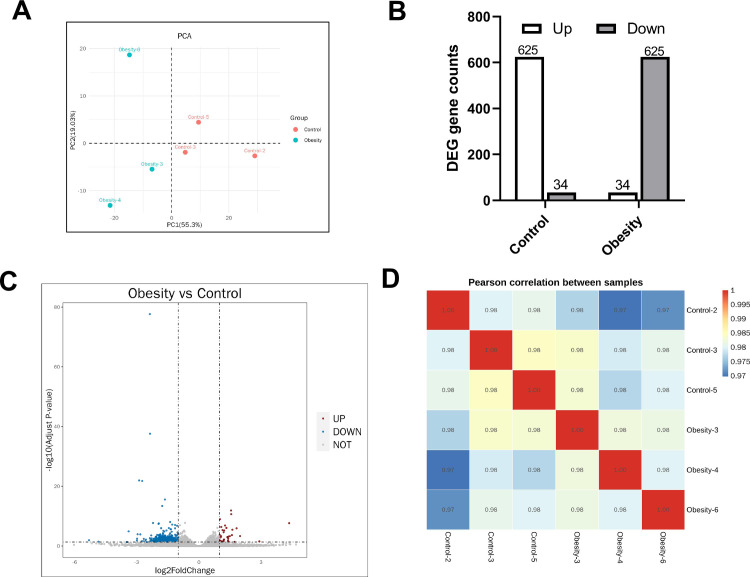
Transcriptomic changes of kidney in obese mice induced by high-fat diet. (A) Principal Component Analysis (PCA). (B) The number of different genes between the two groups. (C) Volcanic map of differential gene expression distribution. (D) Heat map of correlation coefficient between two groups of mouse kidney samples. A-D, n = 3 each group.

Principal component analysis revealed clustering of samples within groups, indicating strong replication (Fig 5A). We identified DEGs using | log_2_FC | ≥ 1 and *P-*adj ≤ 0.05 as criteria. RNA-Seq results showed that 625 DEGs were considerably upregulated and 34 DEGs were substantially downregulated in the control group; the obese group exhibited a contrasting trend (Fig 5B). A volcano plot further illustrated the distribution and expression levels of the downregulated DEGs (Fig 5C). A high correlation coefficient (≥ 0.97) between samples within the same group confirmed the reliability of the results (Fig 5D).

### GO and KEGG enrichment analyses of DEGs

**Fig 6:**
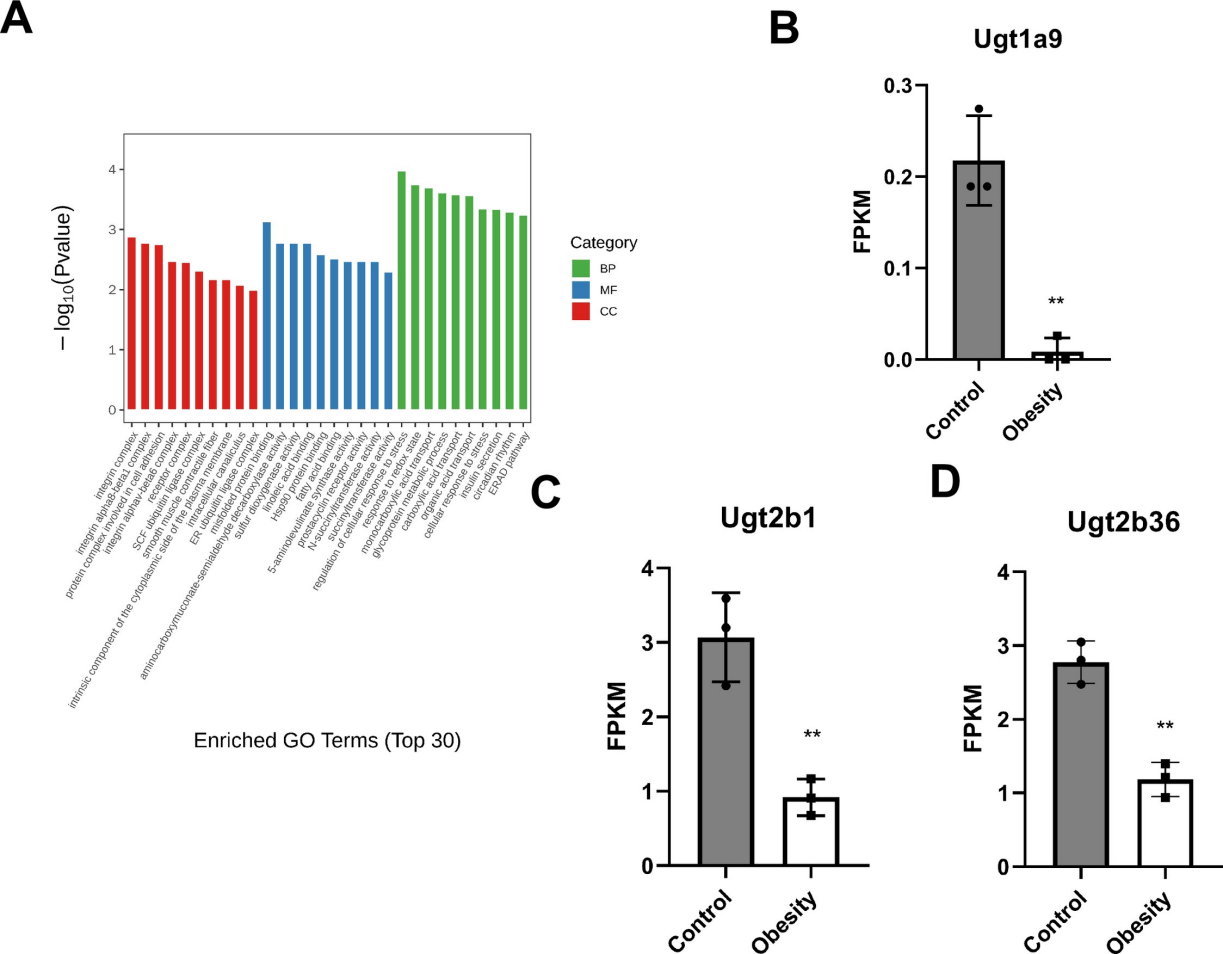
GO enrichment pathway and FPKM of differential genes expression. (A) GO enrichment pathways of upregulated differential genes (B)The FPKM of *Ugt1a9* gene. (C) The FPKM of *Ugt2b1* gene. (D) The FPKM of *Ugt2b36* gene. A-E, **P < 0.01, n = 3 each group.

GO and KEGG enrichment analyses were performed to determine the biological significance of the DEGs. The top 30 highly enriched GO terms associated with cellular components (CC), biological processes (BP), and molecular functions (MF) were identified for upregulated DEGs (Fig 6A). These DEGs were primarily enriched in cellular response to stress and organic acid transport. The significantly enriched GO terms were mainly related to BP.

KEGG enrichment analysis provided insights into renal metabolic changes induced by a high-fat diet in mice. Significant enrichment was observed in the down- and up-regulated metabolic pathways, with a smaller *P*-value indicating greater enrichment (KEGG enrichment revealed one upregulated and four downregulated metabolic pathways; Table 1). The KEGG enrichment revealed disruptions in metabolic pathways in obese mice. We observed a trend of ascorbate and aldarate metabolism pathway downregulation. In the ascorbate and aldarate metabolism pathway, *Ugt1a9* (UDP glucuronosyltransferase 1 family, polypeptide A9), *Ugt2b1*(UDP glucuronosyltransferase 2 family, polypeptide B1), and *Ugt2b36* (UDP glucuronosyltransferase 2 family, polypeptide B36) were involved. The FPKM of these three genes in the kidneys of obese mice was significantly lower than that of the control group (Fig 6B–6D).

**Table 1:** Transcriptomic analysis metabolism-related pathways by KEGG enrichment. (Obesity vs Control).

Pathway	*P* value	FDR	richFactor	Gene	Trend
Histidine metabolism	0.058531345	0.37014404	0.041666667	*Aldh3b3*	NS
Tyrosine metabolism	0.093453997	0.37014404	0.025641026	*Aldh3b3*	NS
Phenylalanine metabolism	0.053780127	0.37014404	0.045454545	*Aldh3b3*	NS
Tryptophan metabolism	0.116052132	0.37014404	0.020408163	*Acmsd*	NS
Porphyrin and chlorophyll metabolism	0.093453997	0.37014404	0.025641026	*Alas2*	NS
Sulfur metabolism	0.027241019	0.37014404	0.090909091	*Ethe1*	up
Metabolism of xenobiotics by cytochrome P450	0.155394173	0.37014404	0.014925373	*Aldh3b3*	NS
Drug metabolism ‐ cytochrome P450	0.151105934	0.37014404	0.015384615	*Aldh3b3*	NS
Metabolic pathways	0.32596031	0.423472536	0.003298153	*Alas2*, *Ldc1*, *Acmsd*, *Aldh3b3*, *Ethe1*	NS
Ascorbate and aldarate metabolism	0.050005619	0.242334923	0.107142857	*Ugt1a9*,	down
				*Ugt2b1*,	
				*Ugt2b36*	
Steroid hormone biosynthesis	0.00795719	0.062662874	0.090909091	*Cyp2c29*,	down
				*Cyp3a11*,	
				*Cyp1a2*,	
				*Gm10681*,	
				*Ugt1a9*, *Ugt2b1*, *Ugt2b36*	
Caffeine metabolism	0.008401227	0.064154827	0.4	*Cyp1a2*, *Uox*	down
Fatty acid metabolism	0.035181009	0.184700297	0.081967213	*Acsbg1*, *Scd1*, *Fasn*, *Acsl6*, *Acaca*	down

NS:No significant difference

### Network analysis of differential metabolites and genes

To determine the number of metabolic pathways linked to the obesity, we compared the pathways associated with DEGs in the transcriptome with differentially expressed metabolites. Differentially expressed metabolites were involved in 177 pathways, DEGs in 119 pathways, and both differential metabolites and genes in 77 pathways (S1-3 Fig in S1 File). The top 10 KEGG pathways with the highest number of co-involved genes and metabolites were primarily within metabolic pathways, with 314 metabolites and 11 genes identified (S1-4 Fig in S1 File). Pathway enrichment analysis of transcriptome and metabolome data confirmed that the ascorbate and aldarate metabolism pathways were the most enriched (S1-5 Fig in S1 File).

### Ascorbate and aldarate metabolism change under obese mice kidney tissue

**Fig 7:**
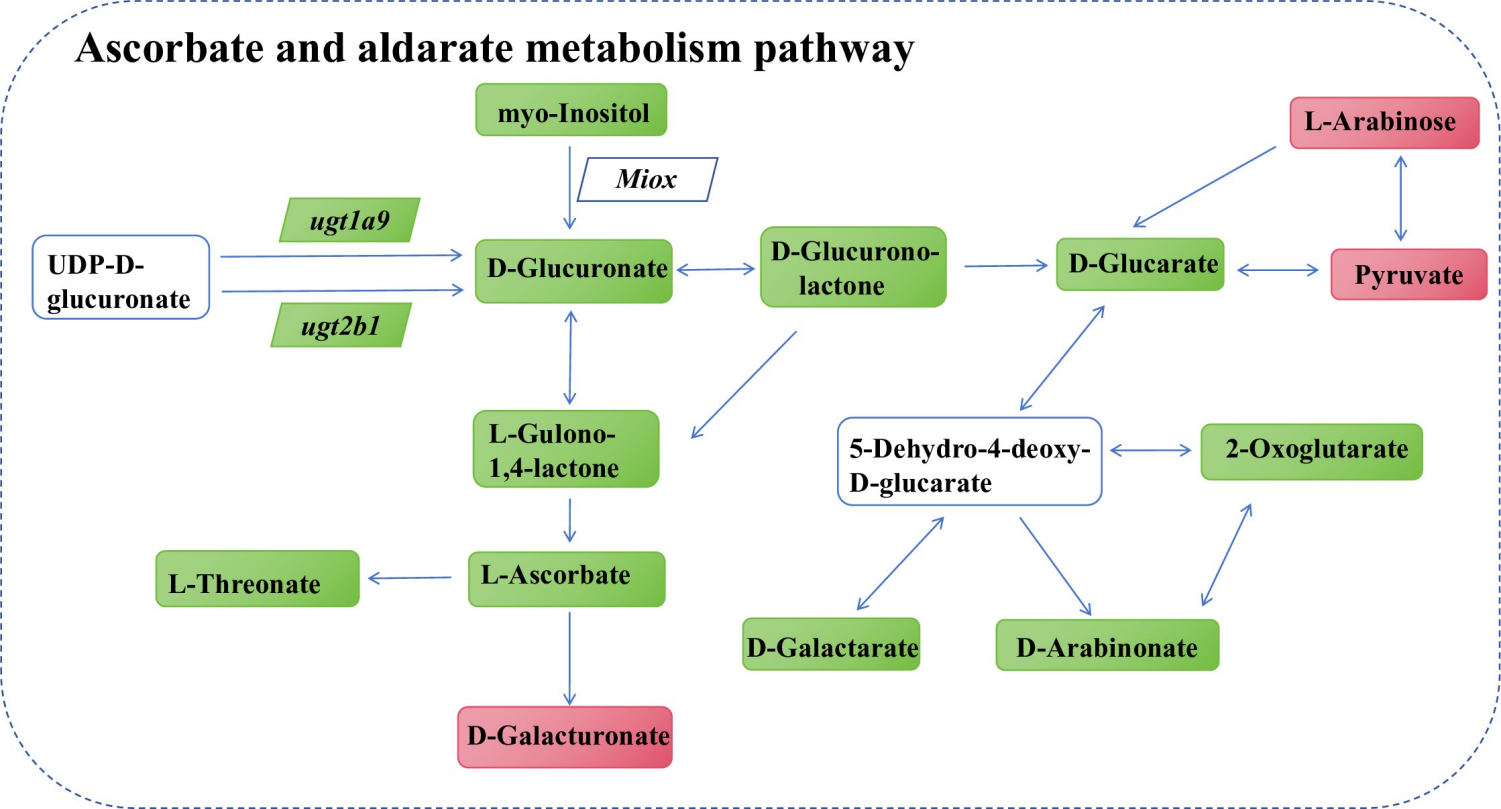
Network analysis of differential metabolites and genes ascorbate and aldarate metabolism pathway. Parallelogram represents differential genes, rectangle represents differential metabolites, green means down-regulated, red means up-regulated.

Ascorbate is a powerful active antioxidant that confers protection against excess ROS. From UDP-D-glucuronate to D-glucuronate, *Ugt1a9* and *Ugt2b1* genes are involved, the expression of *Ugt1a9* and *Ugt2b1* was downregulated, and the content of D-glucuronate was decreased in obese kidney, suggesting that obesity can affect ascorbate biosynthesis (Fig 7). Moreover, conversion of myo-inositol to D-glucuronate requires the participation of myo-inositol oxygenase (mixo), and decrease in the level of myo-inositol lead to a decrease in D-glucuronate. L-gulono-1,4-lactone is the direct precursor of ascorbate. Decreases in the levels of the two metabolites, D-glucuronate and D-glucurono- lactone, leads to a decrease in L-gulono-1,4-lactone, which in turn decreases the ascorbate level. Pyruvate and L-arabinose indirectly affect ascorbate metabolites, and the increase in the pyruvate and L-arabinose content in kidney tissues of mice with high-fat diet-induced obesity indirectly led to a decrease in the ascorbate content (Fig 7). The correlation analysis network of differential genes and metabolites in the ascorbate and aldarate metabolism pathway is shown in S1-6 Fig in S1 File. However, the expression changes in metabolites and genes in kidneys injured via high-fat diet intake in obese mice were not completely consistent, and may be related to other unknown factors.

**Fig 8:**
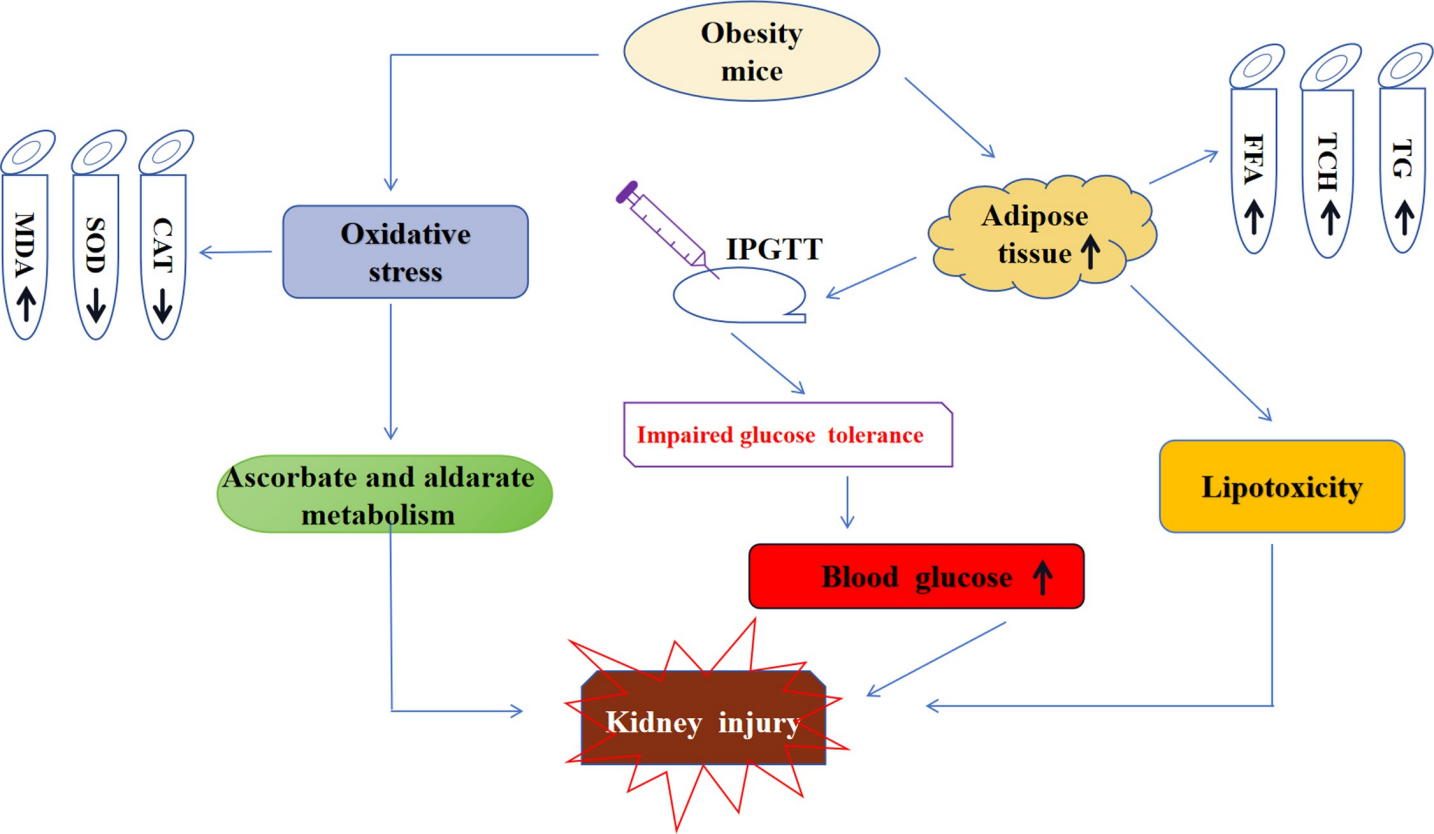
The metabolic characteristics and key biological pathways of kidney injury induced by high-fat diet in obese mice.

This study explored the metabolic characteristics and key biological pathways of kidney injury induced by high-fat diet in obese mice (Fig 8).

## Discussion

This study provides valuable insights into the effects of a high-fat diet on obesity, highlighting abnormal glucose metabolism, increased kidney lipid content, serum free fatty acid (FFA) levels, and visceral fat accumulation, which collectively contribute to renal injury, oxidative stress, and alterations in the ascorbate and aldarate metabolism pathway.

Specifically, mice fed on a high-fat diet for 12 weeks exhibited elevated circulating FFAs and abnormal lipid accumulation in the kidneys. This observation aligns with the previous research that demonstrates how energy intake exceeding the body’s adipose tissue storage capacity leads to the release of circulating lipids into non-adipose tissues such as muscle, liver, and kidneys tissues [38]. Obesity accelerates lipolysis within adipose tissue, increasing FFAs in the bloodstream, which flow to peripheral tissues and lead to lipotoxicity [39]. The accumulation of FFAs, triglycerides, and toxic metabolites in non-adipose tissues can cause renal lipotoxicity, resulting in kidney damage [40, 41]. High-fat diets can lead to increased FFA uptake, upregulation of fatty acid uptake systems (such as CD36 scavenger receptors and fatty acid transporters), and suppression of beta-oxidation rates, thus contributing to intracellular lipid accumulation in non-adipose tissue [42]. Moreover, overexpression of lipogenic enzymes and inhibition of lipid breakdown leads to excessive lipid deposition in the kidneys. Ascorbate is closely related to cholesterol metabolism and can assist in the conversion of cholesterol into bile acids to help reduce cholesterol content. The decrease in ascorbate in kidney tissue observed in our investigation may impact conversion of cholesterol into bile acid and increase the cholesterol content. Additionally, chronic hyperglycemia and hyperlipidemia associated with metabolic syndrome contribute to nutrient overload in the kidneys, leading to ectopic lipid accumulation [43]. Components of metabolic syndrome, such as visceral obesity, hyperglycemia, and dyslipidemia [44], have a profound impact on kidney pathophysiology and increase the risk of chronic kidney disease [45–47]. In the present study, PAS staining revealed glomerular sclerosis and tubular damage in obese mice in addition to brush boundary destruction and epithelial cell shedding in the renal tubule region.

Under normal conditions, tubular epithelial cells maintain redox balance by employing an antioxidant stress system to remove excess ROS. Fat accumulation in the kidneys accelerates inflammation, oxidative stress, and other forms of kidney damage [48]. Collectively, all cellular injury mechanisms related to lipotoxicity, including oxidative stress, can cause kidney injury [49]. Under obese conditions, the kidneys are at a risk of cellular stress, especially with heightened ROS levels and enhanced inflammation [50]. Hyperglycemia leads to the production of numerous oxygen free radicals and lipid peroxidation products through autooxidation and glycosylation end products, causing damage to various organs, including the kidneys [51]. The toxic effects of lipids observed in this study were reflected in heightened oxidative stress. Excess FFAs can damage podocytes, proximal tubule epithelial cells, and tubule interstitial tissue via promoting ROS production and lipid peroxidation, leading to mitochondrial damage and tissue inflammation, eventually contributing to kidney injury [42].Extensive oxidative metabolism in the body produces a large amount of oxygen free radicals. Ascorbate can preferentially bind to these reactive oxygen species, transforming ascorbate into monodehydrogenated ascorbate to exert its antioxidant effects. In our study, the increase of oxidative stress and the decrease in ascorbate levels led to the weakening of the antioxidant system and the aggravation of kidney injury. The kidney tissue of obese mice exhibited reduced basement membrane thickness, blurred edges, uneven thickness, partial foot process fusion, abnormal podocyte morphology and structure, swollen mitochondria, and fractured cristae.

Ascorbate can be synthesized *de novo* via the hexuronic acid pathway in the liver or kidneys of organisms endowed with gulonolactone oxidase activity [52]. Ascorbate is metabolized in the liver and kidneys and is related to carbohydrate metabolism [52]. In this study, the fat content of the diet fed to experimental mice was high and the carbohydrate content was relatively low, which may affect the metabolism of ascorbic acid. Ascorbate, as a powerful water-soluble antioxidant, exerts various biochemical effects, protects tissues from oxidative products, and maintains the reduced state of some enzymes, together with scavenging free radicals and monomer oxygen. Furthermore, ascorbic acid removes oxygen free radicals to stabilize mitochondrial membranes and reduce Cytochrome C release, thus inhibiting the apoptotic cascade initiated by ischemia-reperfusion injury [53]. Ascorbate competitively inhibits non-enzyme-mediated glycosylation, thereby suppressing protein glycosylation and inhibiting the generation of oxygen free radicals and lipid peroxidation products. Ascorbate also directly reacts with oxygen free radicals and lipid peroxidation products, mitigating the damage caused by high oxidative stress in the kidneys. In our study, the ascorbate level was decreased in kidney tissue of obese mice, which weakened the ability to combat oxidative stress, thus compromising the renal tissue’s antioxidant barrier against free radicals. Ascorbate also protects the glomerular filtration barrier structure and maintains kidney function by reducing MMP-2-mediated basement membrane disruption [54]. It stabilizes the production of tetrahydro sphenoid, a cofactor of nitric oxide synthase, indirectly promoting nitric oxide synthesis, which regulates glomerular vasodilation and increases kidney blood flow, further safeguarding kidney function [55]. Additionally, pretreatment with ascorbate considerably reduces the expression of NF-κB p65 expression in the medulla of renal membrane, inhibiting inflammatory damage mediated by the NF-κB signaling pathway and protecting the renal tissue in a dose-dependent manner [56]. In this study, the ascorbate level in the kidney tissues of obese mice was considerably reduced, indicating exacerbated kidney injury. Oxidative stress in obese mice damages the outermost layer of the glomerular filtration barrier, aggravating kidney damage. The decrease in ascorbate content further diminished the antioxidant capacity of kidney tissue, thereby compounding oxidative stress and kidney injury. In addition, obesity models are time-limited, and future research into the effects of obesity at different points in time on the kidney ascorbate and aldarate metabolism pathway may be helpful.

## Conclusions

Our integrative transcriptome and metabolome analysis successfully identified the regulatory candidates involved in ascorbate and aldarate metabolism that play key roles in oxidative stress injury. Our findings suggest that renal oxidative stress injury induced by a high-fat diet affects the metabolism of the ascorbate and aldarate metabolism pathway in obese mice, and that ascorbate can be used as a biological marker of kidney injury in obesity. These novel insights shed light on the metabolic characteristic underlying obesity-induced kidney damage. Based on our findings, we conclude that ascorbate supplementation can reduce the effects of obesity on kidney injury.

## Supporting information

S1 File (DOC)

S2 File (DOC)
