# Supplementary material for: Elucidating ascorbate and aldarate metabolism pathway characteristics via integration of untargeted metabolomics and transcriptomics of the kidney of high-fat diet-fed obese mice
Source: PLoS One. 2024 Apr 11;19(4):e0300705. doi: 10.1371/journal.pone.0300705 (PMC11008897; doi:10.1371/journal.pone.0300705)
Supplement: S1 File — (DOC) [file pone.0300705.s001.doc]

S1 File


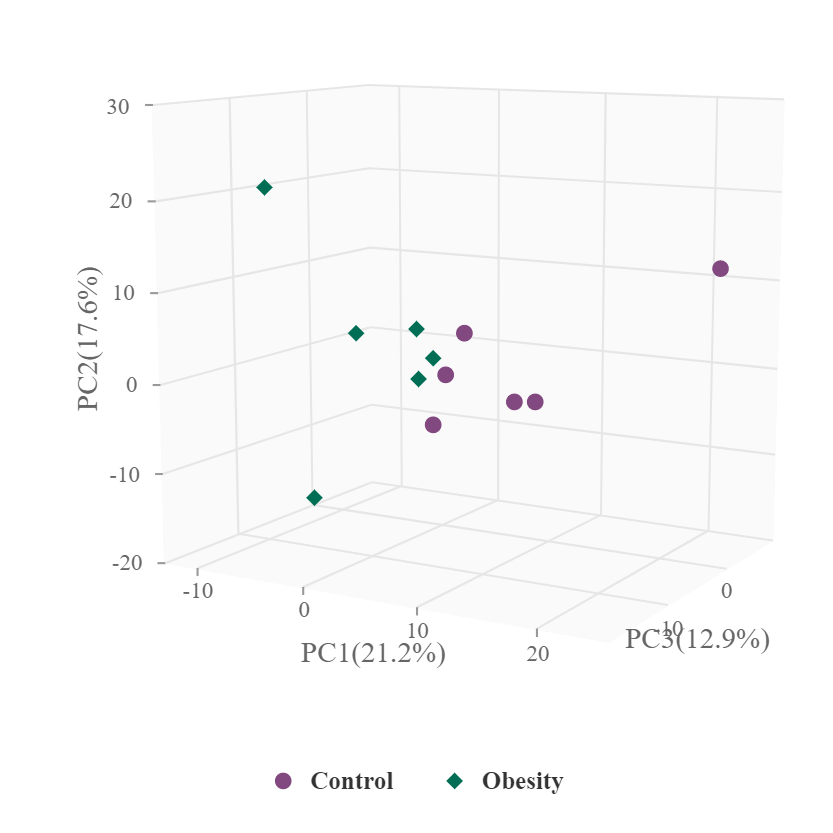


**S1 Fig1.**PCA analysis of positive ion model samples

**
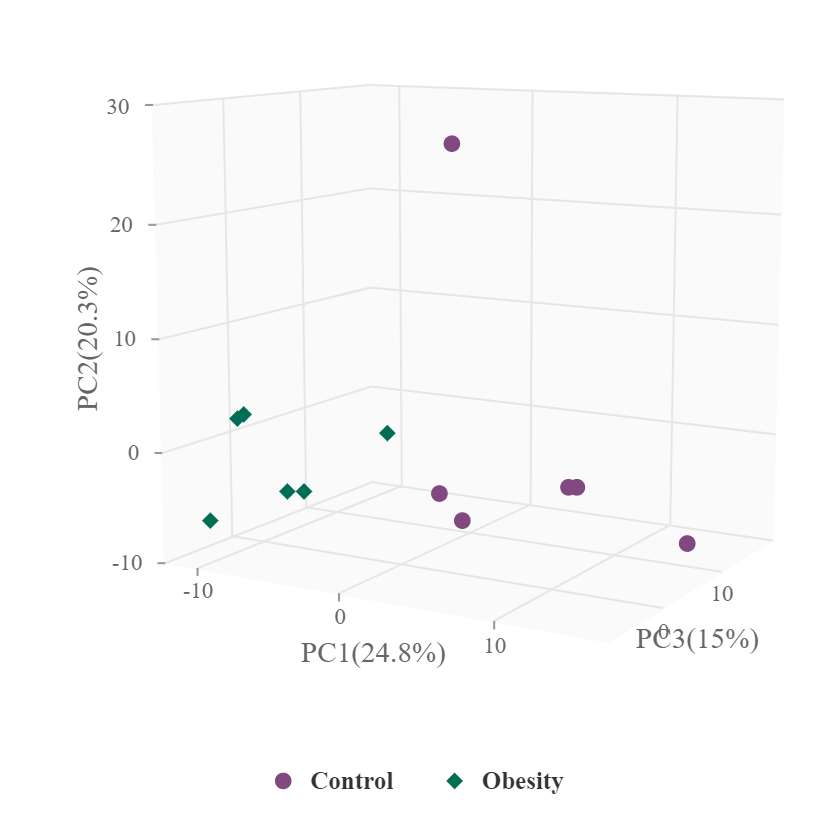
**

**S1 Fig2.**PCA analysis of negative ion model samples


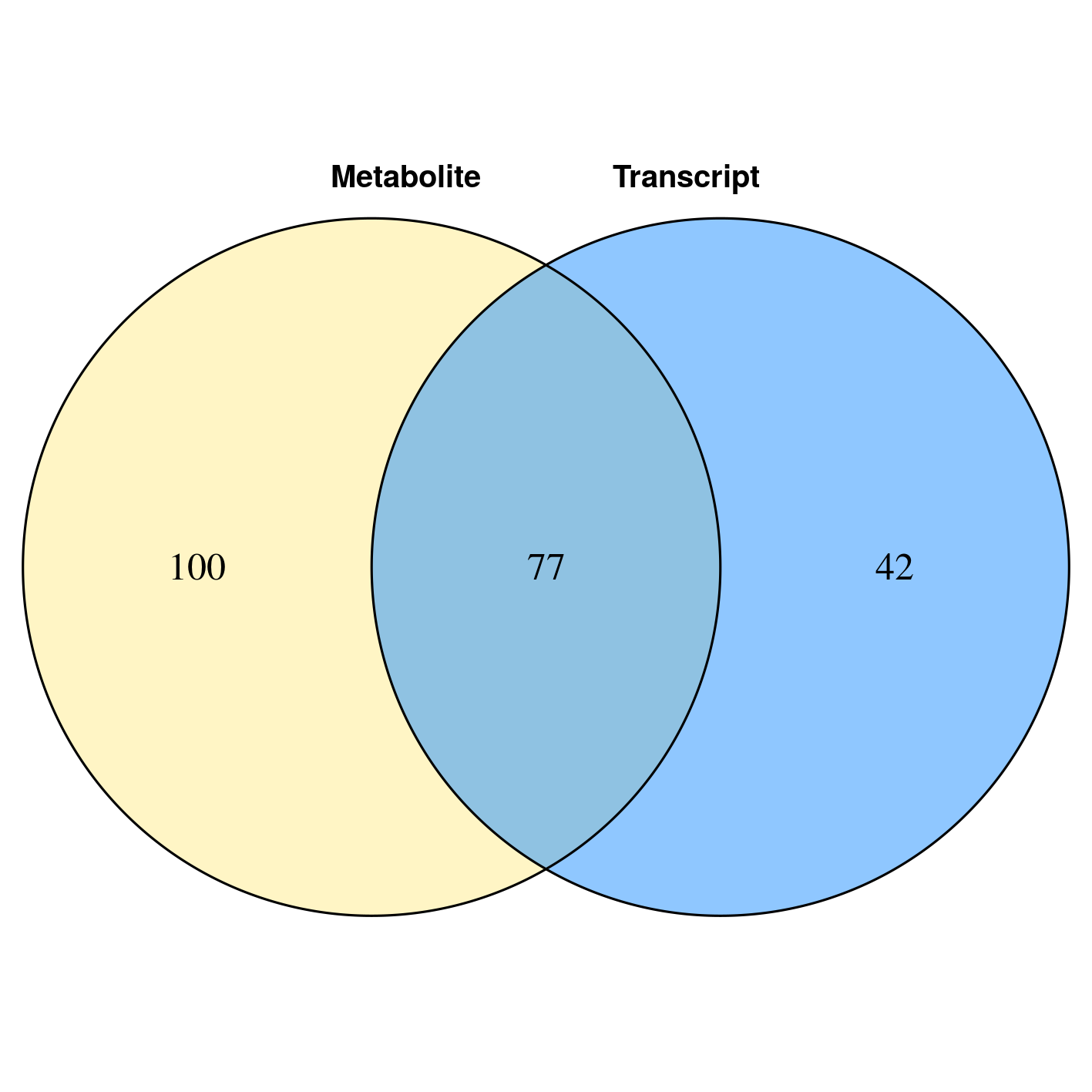


**S1 Fig3.**Venn Diagram of pathways involving differential genes and differential metabolites


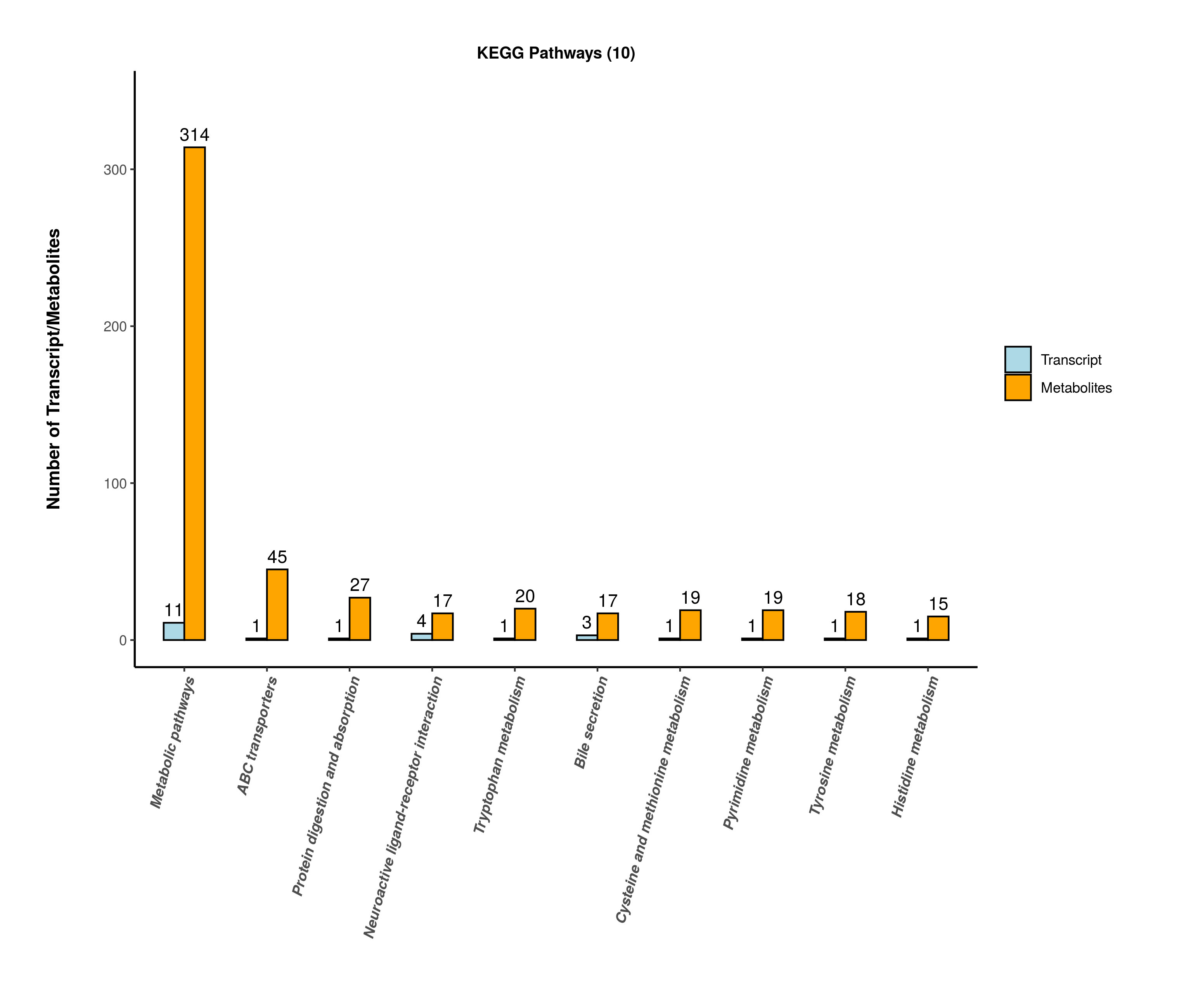


**S1 Fig4.**Genes and metabolites identified in this study were involved in 10 of the top pathways from KEGG


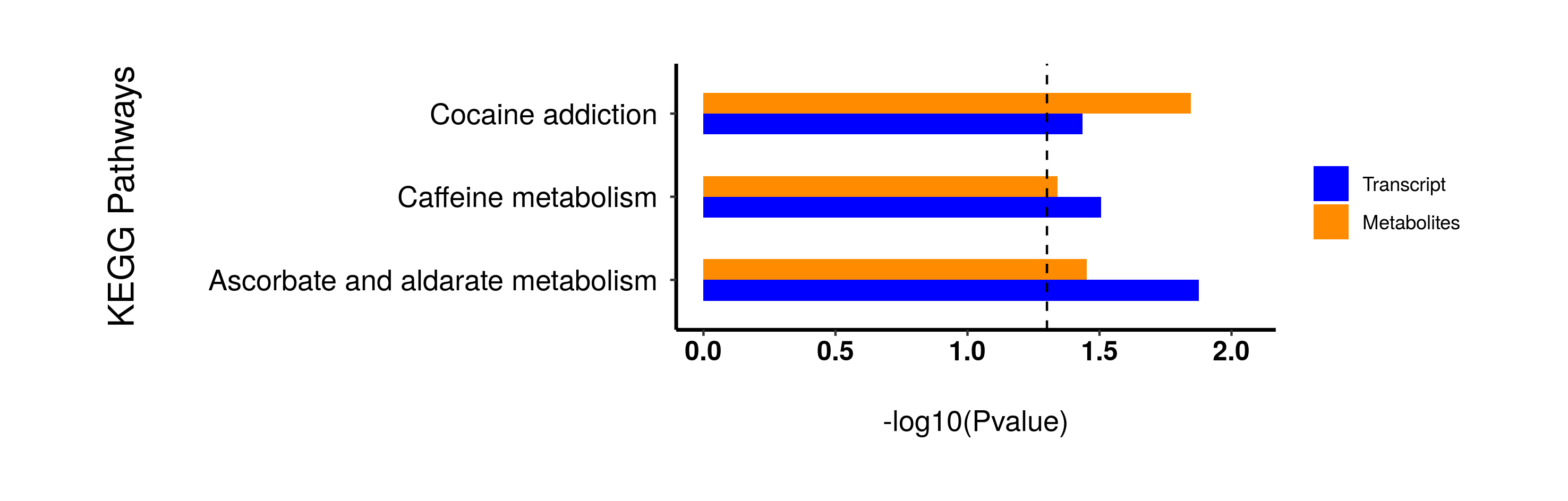


**S1 Fig5.**Histogram of differential gene and metabolite KEGG enrichment

**
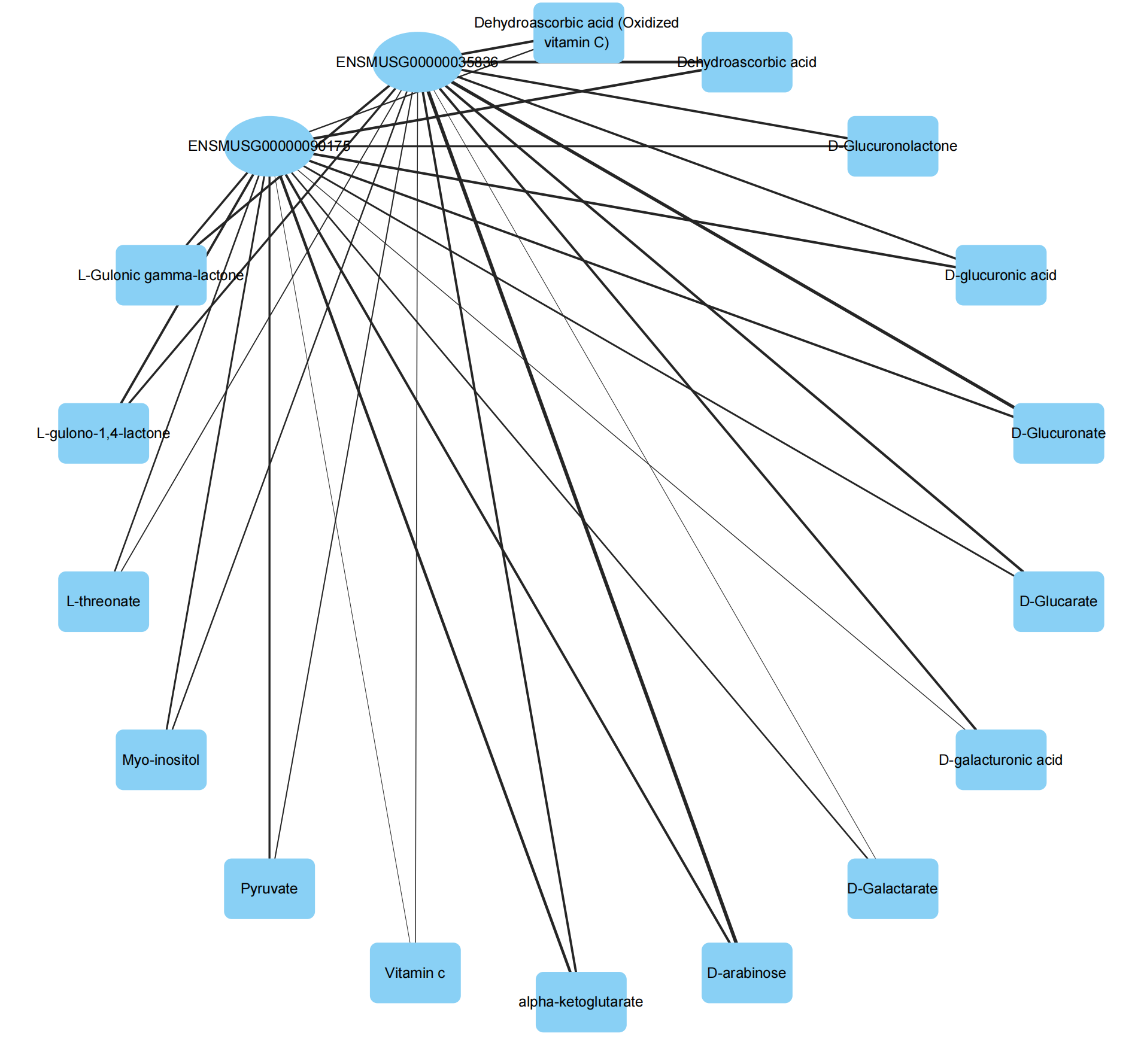
**

**S1 Fig6.** Correlation networks of differential genes and metabolites in ascorbate and aldarate metabolism pathway. Ellipsoid represent gene,rectangle represent metabolite
