# Supplementary material for: Elucidating ascorbate and aldarate metabolism pathway characteristics via integration of untargeted metabolomics and transcriptomics of the kidney of high-fat diet-fed obese mice
Source: PLoS One. 2024 Apr 11;19(4):e0300705. doi: 10.1371/journal.pone.0300705 (PMC11008897; doi:10.1371/journal.pone.0300705)
Supplement: S2 File — (DOC) [file pone.0300705.s002.doc]

Supplementary Material 2

**S2 Table 1 Differential metabolites detected in kidney of obese mice by metabolomic analysis in** **ascorbate and aldarate metabolism pathway (obesity group vs control group)**

| **metabolites** | **rt(s)** | **SuperClass** | **Class** | ***p*-value** |
| --- | --- | --- | --- | --- |
| Pyruvate | 321.682500 | Organic acids and derivatives | Keto acids and derivatives | 0.111866489 |
| alpha-ketoglutarate | 330.592000 | Organic acids and derivatives | Keto acids and derivatives | 0.0842722 |
| Vitamin c | 390.787000 | Organoheterocyclic compounds | Dihydrofurans | 0.045682934 |
| Myo-inositol | 412.972000 | Organic oxygen compounds | Organooxygen compounds | 0.186546114 |
| D-Glucuronate | 309.710000 | Organic oxygen compounds | Organooxygen compounds | 0.043393666 |
| D-arabinose | 367.947000 | Organic oxygen compounds | Organooxygen compounds | 0.0017729 |
| D-galacturonic acid | 368.701000 | Organic oxygen compounds | Organooxygen compounds | 0.013962115 |
| D-Glucarate | 298.970000 | Organic oxygen compounds | Organooxygen compounds | 0.115066791 |
| D-Galactarate | 210.049500 | Organic oxygen compounds | Organooxygen compounds | 0.26766189 |
| L-Gulonic gamma-lactone | 374.222500 | Organoheterocyclic compounds | Lactones | 0.011750146 |
| L-threonate | 364.422000 | Organic oxygen compounds | Organooxygen compounds | 0.947481132 |
| D-Glucuronolactone | 402.748000 | Organoheterocyclic compounds | Furofurans | 0.69657968 |
| Dehydroascorbic acid | 354.616000 | Organoheterocyclic compounds | Lactones | 0.092803643 |
